# Chronic intestinal pseudo-obstruction secondary to intestinal leiomyositis in a ragdoll cat

**DOI:** 10.3389/fvets.2026.1842980

**Published:** 2026-08-03

**Authors:** Carmen W. W. Lau, Aimee Hope, Emma Donnelly

**Affiliations:** Department of Emergency and Critical Care, VetsNow Referral Hospital Glasgow, Glasgow, United Kingdom

**Keywords:** chronic intestinal pseudo-obstruction, functional obstruction, intestinal dilation, intestinal leiomyositis, leiomyositis

## Abstract

A 1-year-old neutered male neutered Ragdoll cat was presented with a 4-week history of hyporexia, intermittent diarrhoea and weight loss. Initial imaging revealed a varying degree of distension of the intestinal loops by echoic fluid/gas and some loops had hyperechoic content with marked echo shadowing. Exploratory laparotomy was recommended but declined. Subsequent clinical deterioration prompted further investigation with CT scan revealing severe dilation of the intestine which was deemed likely secondary to small intestinal foreign body obstruction, and less likely to be secondary to a condition resulting in functional ileus. Surgical exploration revealed severe intestinal distension extending from mid-jejunum to the caecum and the distended portion of the intestine was impacted with hard faecal matter towards the caecum. Surgical resection of the ileum and caecum was performed. Unfortunately, following surgery the patient continued to decline and was euthansied. On post-mortem examination, histhopathology confirmed myopathic instestinal leiomyositis. To our knowledge, this is the first reported case of myopathic intestinal leiomyositis in a cat. This report supports inclusion of pseudo-obstruction as a differential diagnosis in cats with non-obstructive intestinal disease, highlights the need for further investigation to better characterize intestinal pseudo-obstruction in veterinary medicine, and suggests that insights from human medicine may inform diagnostic and disease classification approaches in the interim.

## Introduction

An acute presentation of collapse with chronic gastrointestinal signs is a common presentation to veterinary emergency hospitals, caused by various etiologies. Chronic intestinal pseudo-obstruction (CIPO) is rare and suspected to be underreported in canine and feline medicine. Previously reported causes of CIPO in cats include muscle-actin alpha deficiency, ganglioneuritis and visceral myopathy. This is the first peer-reviewed case of intestinal leiomyositis and CIPO in a Ragdoll cat.

## Case description

A one-year-old male neutered Ragdoll with no previous medical history presented to a primary care veterinarian with a four-week history of hyporexia, intermittent diarrhoea and weight loss. On presentation the patient was collapsed with palpation of an abdominal mass; the cat was subsequently referred to our Out-of-Hours’ service (Supplementary Table 1).

The initial workup identified a moderate anaemia (Hb 5.6 g/dL, RI 9–17, Hct 0.119 L/L, RI 0.303–0.523), mild neutrophilia (25.70 × 10^9^, RI 2.87–17.02), moderate lymphocytosis (11.57 × 10^9^/L, RI 0.92–6.88), mild monocytosis (2.29 × 10^9^/L, RI 0.05–0.67) and mild thrombocytopenia (85 × 10^9^/L, RI 151–600). Biochemistry identified mild total hypocalcaemia (1.85 mmol/L, RI 1.95–2.83), reduced ALP (<10 U/L, RI 14–111), mild increase in GGT enzyme activity (23 U/L, 0–4), decreased amylase (226 U/L, RI 500–1,500) and mildly decreased lipase (98 U/L, RI 100–1,400).

Repeat external haematology and biochemistry confirmed a moderate non-regenerative anaemia (Hb 4.8 g/dL, RI 9–17, HCT 14%, RI 27–50) and mild neutrophilia (19.52 × 10^9^, RI 2.5–12.5), however platelet manual count was higher, approximately 160–180 × 10^9^/L. Biochemistry identified mild hypoalbuminaemia (22 g/L, RI 25–42), mild total hypocalcaemia (1.78 mmol/L, RI 2.0–3.0), mildly reduced creatinine (51 μmol/L, RI 80–180), mild hyperbilirubinaemia (19.3 μmol/L, RI 0–10), moderately increased bile acids (63.1 μmol/L, RI 0–10) and severely increased creatine kinase (838 U/L, RI 0–152).

This patient was referred to our Internal Medicine specialist service and had further investigation; results of which included a negative in-house saline agglutination test, negative Coombs’ test and negative feline haemoplasmas qPCR. Repeat external haematology and biochemistry confirmed a moderate non-regenerative anaemia (Hb 4.8 g/dL, RI 9–17, HCT 14%, RI 27–50) and mild neutrophilia (19.52 × 10^9^, RI 2.5–12.5), however platelet manual count was higher, approximately 160–180 × 10^9^. Biochemistry identified mild hypoalbuminaemia (22 g/L, RI 25–42), mild total hypocalcaemia (1.78 mmol/L, RI 2.0–3.0), mildly reduced creatinine (51 μmol/L, RI 80–180), mild hyperbilirubinaemia (19.3 μmol/L, RI 0–10), moderately increased bile acids (63.1 μmol/L, RI 0–10) and moderately increased creatine kinase (838 U/L, RI 0–152). Abdominal ultrasonography identified varying degrees of distension of the intestinal loops by echoic fluid/gas; some loops (which were markedly firm on external palpation) had hyperechoic content with marked echo shadowing which was thought to represent faeces, trichobezoar or foreign material. It was challenging to determine whether they were small or large intestinal loops. The walls of the intestinal loops were hypoechoic and layering was blurred but not thickened. Peristalsis was reduced; distal part of the descending colon was empty. There was moderate peritoneal effusion and moderate to marked mesenteric lymph node enlargement.

Due to concerns of either intestinal obstruction or functional disease, and given the severity of the patients’ condition, an exploratory laparotomy was recommended. The decision was made by a proxy owner to hold surgical intervention and continue medical management.

An in-house examination of cytology did not find any evidence of intracellular bacteria and analysis with blood glucose and lactate was not consistent with a septic exudate. External pathologist-reviewed cytology of the peritoneal effusion was consistent with a low cellularity modified transudate with occasional nucleated cells consisting of macrophages along with less frequent neutrophils and lymphocytes. Low numbers of mast cells were also noted. 3 days later we received interim pleural effusion culture results which revealed Acinetobacter species, sensitive to amoxicillin, amoxicillin-clavulanic-acid and marbofloxacin. This was consistent with the extended culture results and the pathologist has noted could be a potential contaminant.

Thirty-six hours post-admission, prior to return of the culture report, the patient was internally transferred to the Emergency and Critical Care specialist service. The treatment to that point included analgesia, dexamethasone (single dose at referring vet), fluid therapy, maropitant, and canine xenotransfusion. Due to the unavailability of stored feline blood, the risks and benefits of xenotransfusion were discussed and accepted by the owners. Cat blood donors were available to be contacted should another transfusion be required. In the following days, given the chronicity of this patient’s condition, with no parameters of transfusion dependency, and stability in PCV and HCT there was no indication for further transfusion.

On presentation, the patient was dull and non-ambulatory. Mucous membranes were pale pink and moist with capillary refill time of 1.5 s. No petechiae or ecchymoses were present. Heart rate was 120 beats per minute with strong and synchronous peripheral pulses. No murmur was noted on auscultation. Doppler blood pressure showed hypotension (systolic blood pressure 63 mmHg). Respiratory rate was 28 breaths per minute. There was normal respiratory effort, and no adventitious lung sounds were detected. A markedly distended abdomen with distended abdominal loops without palpable masses was noted. Peripheral lymph nodes were unremarkable. Rectal temperature was 35.3 °C. Body condition score was 2/9 with body weight of 2.87 kg. Fluorescein dye test revealed bilateral superficial corneal ulcers. Corneal ulceration likely occurred due to a combination of opioid treatment and recent general anaesthesia. There was horizontal nystagmus with left fast phase. Overall assessment was consistent with hypovolaemic, distributive shock.

**Figure 1:**
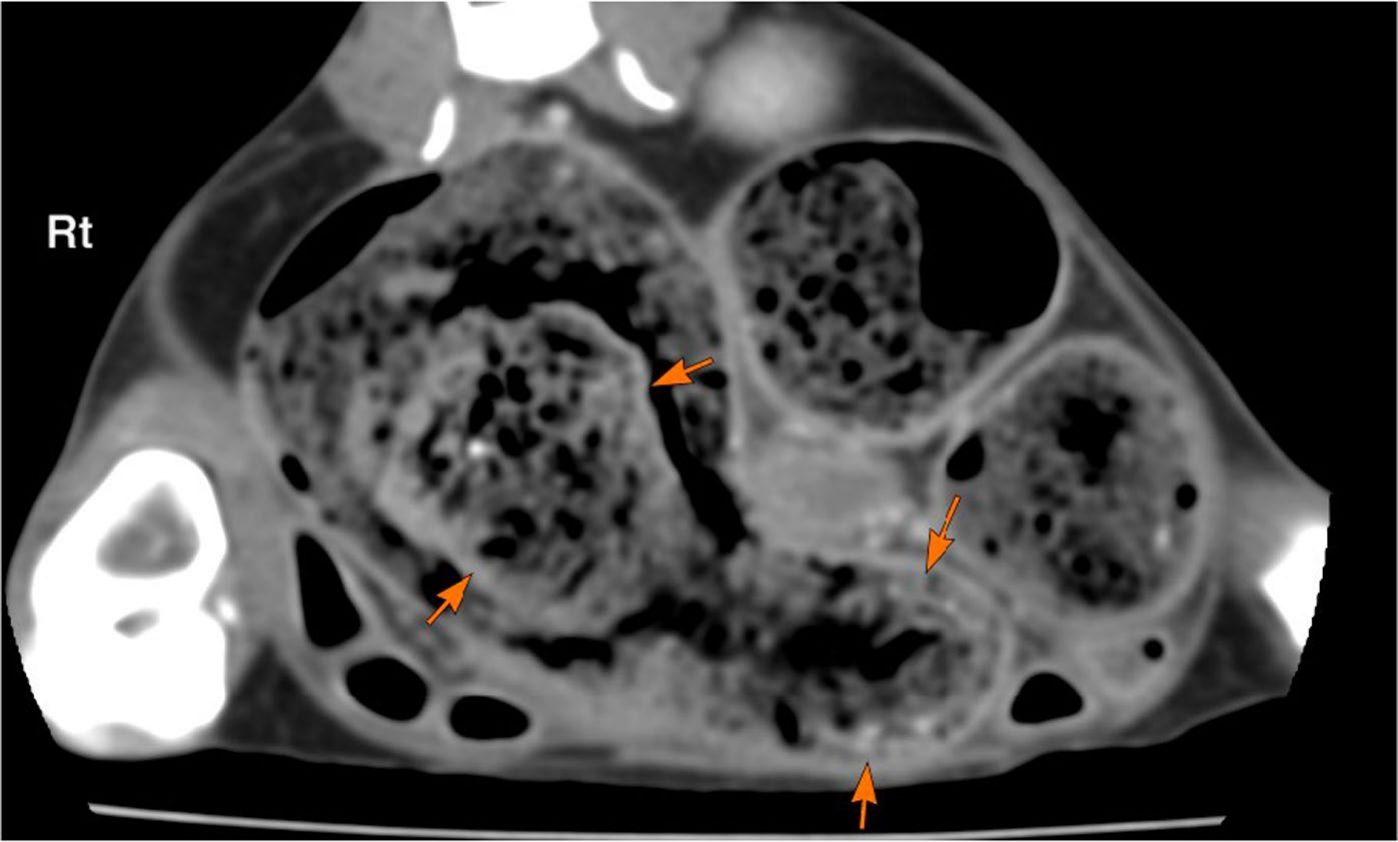
CT sagittal section of the suspected foreign body as represented by the orange arrows. Adapted from Di Nardo et al (1).

A computed tomography scan of the thorax and abdomen was interpreted by a Diagnostic Imaging specialist. The conclusion revealed “a tubular structure passing towards the left-ventral abdomen which was suspected to represent an intestinal foreign body (Figure 1 orange arrows on attached screenshot). The lack of a generalised dilation of the gastrointestinal tract is why a functional ileus, which is generally more of a diffuse process, was considered less likely.” Given these findings, our differentials included functional intestinal obstruction, mechanical intestinal obstruction, neoplasia, vascular event, with the latter two less unlikely.

An exploratory laparotomy was performed which revealed severe intestinal distension extending from mid-jejunum to caecum. The affected intestine was impacted with hard faecal matter which was solidified aborad. Due to concern for functional pseudo-obstruction with a poor prognosis, euthanasia was recommended. The owners elected to continue treatment and ileocaecal resection was elected due to the severe intestinal distension extending into this area including the area of impaction of hard faecal matter. Due to hypotension during the post-operative phase but while under general anaesthesia, it was elected to recover the patient without an esophageal feeding tube or central line placement.

Pre-operatively, treatment included balanced isotonic crystalloids with potassium chloride supplementation, opioid analgesia and continuous infusion of ketamine and fentanyl. The corneal ulcers were managed with fucidic acid, chloramphenicol ointment and hourly lubrication. Unfortunately, the patient worsened in the following 24 h, requiring vasopressors for hypotension. Euthanasia was elected due to poor prognosis.

Histopathology of the ileo-caeco-colic junction revealed severe distortion of architecture, characterized by marked mucosal attenuation and ulceration with inflammatory exudate. Most prominently seen in the muscularis propria, as both inner circular and outer longitudinal smooth muscle layers were extensively infiltrated by mixed inflammatory cells and progressively replaced by fibrous connective tissue. Smooth muscle bundles were disorganised and separated by cellular, vascular fibrous tissue. Inflammatory infiltrates consisted predominantly of lymphocytes, plasma cells and macrophages, with scattered neutrophils and dense lymphoid aggregates. Inflammation extended into the submucosa and muscularis mucosa, causing further architectural distortion. Myenteric plexuses were preserved without evidence of ganglion cell degeneration. Changes were most severe in the ileum, with possible extension into the caecum and colon. Histopathology was submitted to the laboratory prior to euthanasia and therefore no further testing was performed on the samples after euthanasia.

**Figure 2:**
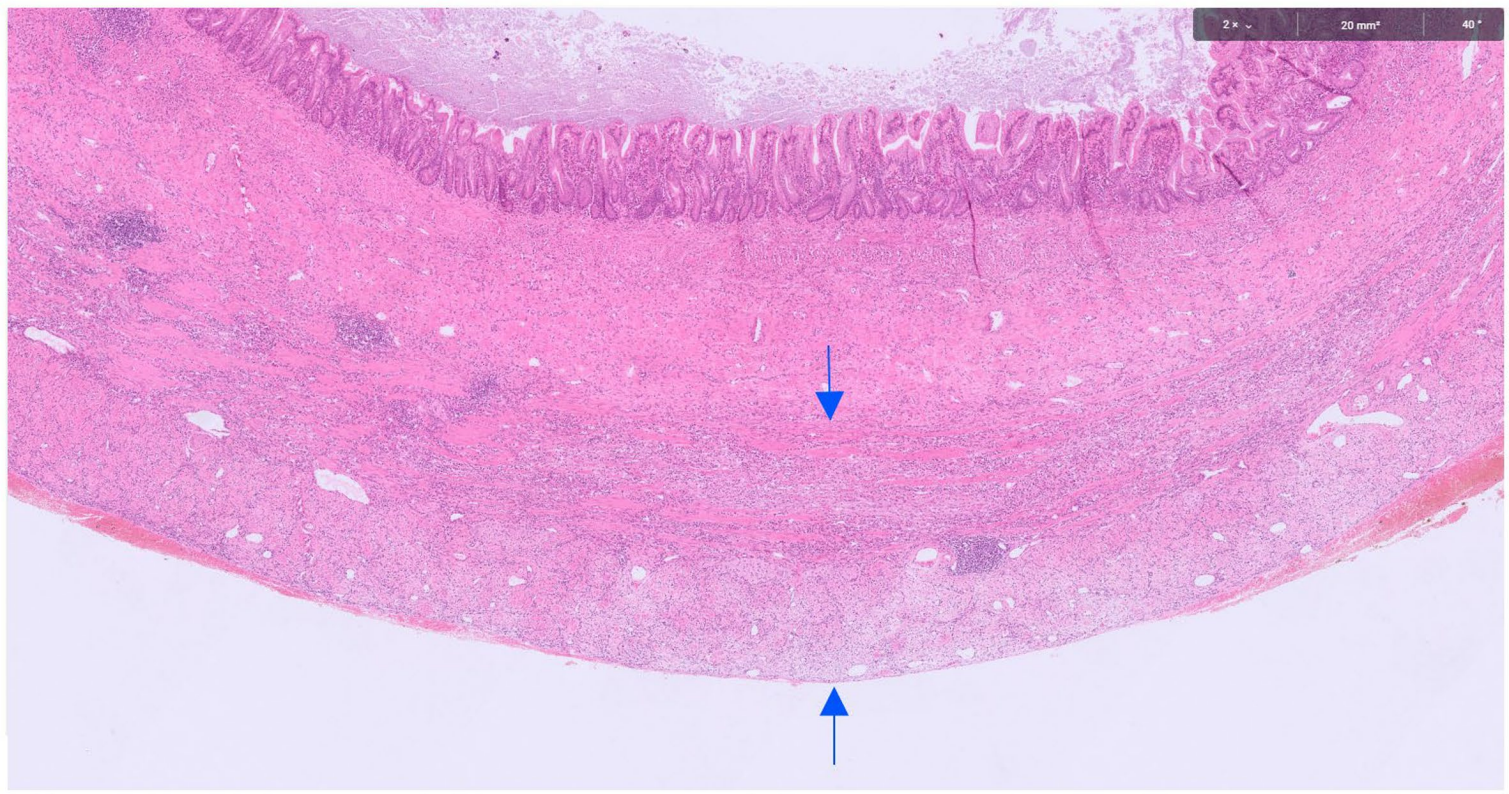
Low power view showing attenuation of the muscular wall of the intestine (between arrows) with loss of distinction between the circular and longitudinal muscle layers. Adapted from Di Nardo et al (1).

**Figure 3:**
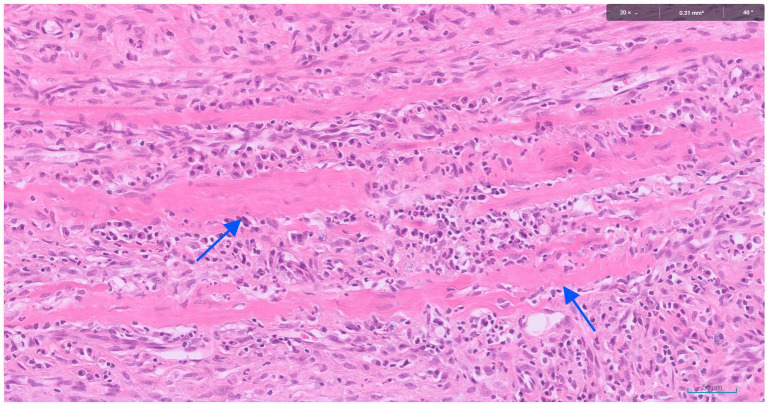
Higher power view with chronic infiltrates of inflammatory cells dissecting between and disrupting the smooth muscle cells. Residual smooth muscle is highlighted with arrows. Adapted from Di Nardo et al (1).

The histopathology findings were consistent with intestinal leiomyositis; severe muscular inflammation with degeneration and necrosis with myocyte atrophy and replacement by fibrosis (Figures 2, 3).

## Discussion

Chronic intestinal pseudo-obstruction (CIPO) is a rare, severe gastrointestinal neuromuscular disorder characterized by failure of the gastrointestinal tract to propel contents in absence of mechanical obstruction (1, 2).

Veterinary literature on CIPO is limited, with the largest case study of 6 dogs (3). In cats, the literature was confined to three case reports, where CIPO has been linked to other pathologies; visceral myopathy (4), ganglioneuritis (5), and muscle alpha-actin deficiency (6).

In human medicine, the European Society for Clinical Nutrition and Metabolism (ESPEN) group (7) specifies dysmotility as the central characteristic of CIPO, which we observed in this patient. It categorizes it into distinct types of neuropathies or myopathies (8) via histopathology. Intestinal leiomyositis (myopathic CIPO) is an inflammatory condition characterized by lymphocytic infiltration within the muscularis propria and with prolonged chronicity leading to secondary fibrotic changes (9). CIPO is additionally broadly classified into two categories: (1) familial or primary CIPO, where no underlying disorder can be identified, and (2) secondary CIPO, associated with underlying gastrointestinal or systemic diseases (10).

Table 1 classification system for chronic intestinal pseudo-obstruction (1).

**Table 1:** Etiology and classification of CIPO.

Primary	Secondary	Familial forms
No demonstrable etiopathogenetic causes	Neurological disorders	Autosomol dominant
	Metabolic diseases	SOX 10^*^
	Paraneoplastic syndromes	Autosomol recessive
	Neurotropic viruses	RAD21^*^
	Autoimmune disorders	SGOL1^*^
	Celiac disease	TYMP^*^
	Neuro-muscular disorders	POLG^*^
	Radiation enteritis	X-Linked
	Endocrinological disorders	FLNA^*^
	Drugs	LICAM^*^

^*^Mutations to the indicated genes.

FLNA, filamin; L1CAM, L1 cell adhesion molecule; POLG, polymerase DNA gamma; RAD21, cohesin complex component; SGOL1, shugoshin-like 1; SOX10, SRY-BOX 10; TYMP, thymidine phosphorylase; CIPO, chronic intestinal pseudo-obstruction.

In the human literature, half of documented adult cases are secondary, however the majority of paediatric patients present with primary and congenital disease (2).

It is not currently known which subdivision is more common in our patient group; histopathological diagnosis is complex and poorly understood. In this case there was initial disagreement on the categorisation due to lack of published data on intestinal leiomyositis. Enteric ganglionitis was excluded as diagnosis as there was no evidence the inflammation was targeting intestinal ganglionic plexuses. A final diagnosis of intestinal leiomyositis with associated pseudo-obstruction, consistent with myopathic CIPO (8), was reached. For complete categorizing, investigations would have included immunohistochemistry (i.e., lymphoma), immunoreactivity testing (i.e., smooth muscle-alpha actin) and PCR testing (i.e., feline infectious peritonitis). The client declined further testing as the patient was deceased.

In human medicine CIPO has variable clinical presentation but is suspected in patients with symptoms of intestinal obstruction; vomiting, inappetence, weight loss, progressive abdominal distension and/or pain, and constipation or diarrhoea secondary to bacterial overgrowth (11). In Paediatric Intestinal Pseudo-obstruction (PIPO) urinary tract involvement has been observed in 33–92% of cases with a hypo-contractile detrusor, increased bladder capacity and increased compliance (bladder adynamia) (2). Urinary tract involvement has not been documented in our patient group to date.

Diagnosis of CIPO is achieved with radiological studies to demonstrate dilated bowel with the absence of mechanical obstruction. Contrast with barium has been avoided to prevent leakage of barium into the colon, and in children the preference has been to use water-soluble contrast (2). This has been replaced by enterography with CT (12) or MRI (13) which may be better suited to exclude physical obstruction. In this case, a CT scan with contrast was performed and the changes were consistent with mechanical obstruction, and retrospectively on retrospective evaluation by a Diagnostic Imaging specialist, the diagnosis of leiomyositis would account for the confounding empty orad and aboral portions of the gastrointestinal tract, particularly in the absence of vomiting to account for the empty stomach and duodenum, highlighting the importance of awareness of functional pseudo-obstruction.

Small intestinal dysmotility in humans is diagnosed by antro-duodenal manometry which measures intraluminal pressure changes within organs such as the oesophagus, intestine and colon (14, 15). Manometry would help differentiate between enteric neuropathy (motor activity is disorganised and/or uncoordinated) from enteric myopathy (normal manometric pattern, but with reduced amplitude of contractions).

Definitive diagnosis in both humans and animals is achieved with full thickness gastrointestinal biopsies. Endoscopic biopsies may fail to achieve a definitive diagnosis (16, 17). Severe variability exists in histopathological techniques, obtained by full-thickness biopsy, with no quantitative or qualitative evaluation of enteric neurons. A histopathological diagnosis provides prognostic information and directs specific management. Histological confirmation of inflammatory visceral neuropathy or myopathy is recommended before starting immunotherapies (18, 19). In this case, intestinal leiomyositis, a form of myopathic CIPO, has been identified through history, radiographic changes and histopathologic evidence of lymphocytic infiltration in the muscularis propria which leads to secondary fibrotic changes. CIPO classification systems in animals have not however been determined.

Treatment involves optimising nutritional intake, promoting motility, relieving symptoms of pain/bloat/nausea, and treating bacterial overgrowth (11, 20). In human patients a low-fibre, low-residue and low-fat diet is recommended, avoiding high concentrations of fat reduces delays in gastrointestinal transit. Feeding strategies including oral, enteral or parenteral nutrition tailored to the patient (21). Two-thirds of PIPO patients require nutritional support long-term. Esophageal feeding tube allows enteral feeding at home and in hospital. However, parenteral nutrition is a short-term solution in an acute setting.

Potentially beneficial pro-kinetic drugs include metoclopromide and cisapride, with a variety of other medications trialed in human patients to varying effects (22). Due to the risk of exacerbating dysmotility and narcotic bowel syndrome opioids are avoided where possible (23). In humans understanding the role of gut-brain neuromodulation, of chronic pain, has facilitated use of alternative medications to opioids, such as tricyclic antidepressants, selective serotonin or serotonin-norepinephrine reuptake inhibitors (24).

Antimicrobials have been used to prevent small intestinal overgrowth due to stasis which can lead to improvement in nutritional status, reducing malabsorption and bloating. However, use of antimicrobials for management of gastrointestinal disorders in veterinary patients has been recently widely criticised (25, 26).

Immunomodulation in inflammatory neuropathies and myopathies has been documented in children (27). Intravenous immunoglobulins or methylprednisolone have been used as first-line treatment. In a canine intestinal leiomyositis case study, intravenous immunoglobulin G was used in combination with other medical therapy, with a survival time greater than 2 years (9).

Other immunosuppressive medications, commonly used in veterinary medicine, such as azathioprine and cyclosporin are considered (11), preferably before mural muscular atrophy and fibrosis occur.

Surgery should be avoided where possible to reduce the risk of postoperative worsening of intestinal function (28). In humans with severe CIPO who are refractory to medical treatment, subtotal enterectomy has been proposed (29, 30). Despite the high post-operative morbidity, this has been shown to improve quality of life, increase oral intake, remove the need for suction gastronomy and decrease abdominal pain and vomiting.

Intestinal transplantation, with or without other organs such as the liver or pancreas, remains the only definitive cure for PIPO (31). This has been performed primarily in children with life-threatening severity of disease or where treatment is refractory to medical management.

Despite these interventions, prognosis in humans and veterinary patients remains guarded to poor, with limited reports of median survival time.

## Conclusion

This case highlights the importance of considering functional intestinal diseases as a differential diagnosis in cats presenting with chronic gastrointestinal signs. Chronic intestinal pseudo-obstruction is a functional intestinal disorder which requires careful investigation and histopathological confirmation for accurate diagnosis, treatment and prognostication. Further studies are essential to systematically record CIPO classifications, improve disease categorisation, establish a histopathological diagnostic framework, and evaluate targeted medical management strategies to optimise patient outcomes.

## Data Availability

The original contributions presented in the study are included in the article/Supplementary material, further inquiries can be directed to the corresponding author.
