# Supplementary material for: Chronic intestinal pseudo-obstruction secondary to intestinal leiomyositis in a ragdoll cat
Source: Front Vet Sci. 2026 Aug 3;13:1842980. doi: 10.3389/fvets.2026.1842980 (PMC13480069; doi:10.3389/fvets.2026.1842980)
Supplement: Supplementary file 1 [file Table_1.DOCX]

# Supplementary Table 1

**Timeline of the Case**

| **Symptomatic period** | **Symptoms (prominent)** | **Diagnostics (significant)** | **Treatment** | **Comment (Diagnosis/ Veterinary Care)** |
| --- | --- | --- | --- | --- |
| 4 week period  Presented at referring veterinary practice | - Hyporexia - Intermittent diarrhoea - Weight loss |  | - Dexamethasone 0.2mg/kg IV - Maropitant 1mg/kg IV | - Elected to refer on the same day to Out-of-Hours’ care |
| Day 1 | - Dull, lateral recumbency - Distended intestinal loops on palpation | - PCV 19% | - Isotonic crystalloid fluid therapy (Hartmann’s) 2ml/kg/hr - Methadone 0.2mg/kg IV q4 then Buprenorphine 0.02mg/kg IV q8 | - Overnight stabilisation - Transferred to Internal Medicine specialist service the following morning |
| Day 2 | - Ongoing quiet mentation | - PCV 15% - Abdominal ultrasound revealed severely distended intestinal loops with content - Peritoneal effusion revealed non-septic exudate in-house | - Canine xenotransfusion 20ml (6.9ml/kg) - Then isotonic crystalloid fluid therapy (Hartmann’s) 2ml/kg/hr - Buprenorphine 0.02mg/kg IV q8 - Eating small amounts the same evening after blood transfusion and then developed dull demeanour and hypotension, trialled with crystalloid fluid bolus x2 | - Offered surgical exploration of the abdomen, declined by owners, continued medical management - Transferred to ECC the following morning as per weekend hospital protocol |
| Day 3 | - Progressively dull demeanour - Progressively painful on current analgesia | - Bilateral corneal ulceration - PCV 33% | - Isotonic crystalloid fluid therapy (Hartmann’s) 2ml/kg/hr with potassium supplementation - Fentanyl 2ug/kg/hr CRI - Fucidic acid eye ointment OU q8 | - Weekend stabilisation |
| Day 4 | - Ongoing inappetence | - PCV 30% | - Isotonic crystalloid fluid therapy (Hartmann’s) 2ml/kg/hr - Fentanyl 2ug/kg/hr CRI - Fucidic acid eye ointment OU q8 | - Weekend stabilisation |
| Day 5 | - Developed ataxia, nystagmus with fast phase left | - PCV 30%, TS 64g/L @7am - PCV 35% at 8pm - Hyperbilirubinemia 45 μmol/L, RI 0-15 - CT performed suggestive of mechanical intestinal obstruction - Proceeded to surgical exploration of the abdomen | - Isotonic crystalloid fluid therapy (Hartmann’s) 2ml/kg/hr - Fentanyl 3ug/kg/hr CRI - Ketamine 5ug/kg/min CRI - Chloramphenicol eye ointment OU q6 | - Given findings on surgical exploration, suspect function intestinal disease, full thickness biopsies taken - Euthanasia offered, owner elected to continue medical management |
| Day 6 | - Consistent hypotension overnight requiring noradrenaline | - PCV 32% @7am |  | - Euthanasia elected |

IV- Intra-venous; CRI – Continuous rate infusion; PCV – Packed cell volume
